# Worst Histology‐Based Risk Stratification for Lymph Node Metastasis in Patients With T1b Colorectal Cancer: A Retrospective Pathology‐based Study

**DOI:** 10.1002/deo2.70355

**Published:** 2026-06-04

**Authors:** Satomi Shibata, Shin‐ichiro Horiguchi, Koichi Koizumi, Daichi Sadato, Ryoko Shimizuguchi, Akinari Takao, Jun Nakahodo, Yawara Kubota, Itaru Kubota, Hiroki Kato, Misato Takao, Daisuke Nakano, Kazushige Kawai, Tatsuro Yamaguchi, Tsunekazu Hishima, Toshiro Iizuka

**Affiliations:** ^1^ Department of Gastroenterology, Tokyo Metropolitan Cancer and Infectious Diseases Center Komagome Hospital Tokyo Japan; ^2^ Department of Pathology, Tokyo Metropolitan Cancer and Infectious Diseases Center Komagome Hospital Tokyo Japan; ^3^ Clinical Research and Trials Center, Tokyo Metropolitan Cancer and Infectious Diseases Center Komagome Hospital Tokyo Japan; ^4^ Nishiarai Coloproctology Clinic Tokyo Japan; ^5^ Department of Surgery, Tokyo Metropolitan Cancer and Infectious Diseases Center Komagome Hospital Tokyo Japan; ^6^ Department of Clinical Genetics, Tokyo Metropolitan Cancer and Infectious Diseases Center Komagome Hospital Tokyo Japan

**Keywords:** lymph node metastasis, poorly differentiated adenocarcinoma, risk stratification, T1 colorectal cancer, worst histology

## Abstract

**Objectives:**

Histological subtype is an important pathological factor in risk stratification for lymph node metastasis (LNM) in T1 colorectal cancer (CRC); however, conventional predominant‐type assessment may miss minor poorly differentiated components in T1b CRC. We aimed to develop a T1b‐specific risk stratification framework incorporating a reproducible worst histology (WH) approach.

**Methods:**

We retrospectively analyzed 488 patients with pathologically confirmed pT1b CRC who underwent surgical resection with lymph node dissection. Histological evaluation was performed using three approaches: dominant histology (DH), WH, and WH focusing on poorly differentiated adenocarcinoma (WH‐por). For each approach, independent risk factors for LNM were identified. Patients without these factors were defined as the low‐risk subgroup, and LNM rates were compared with the conventional Japanese Society for Cancer of the Colon and Rectum (JSCCR) criteria.

**Results:**

In patients with T1b colon cancer, the WH‐por approach demonstrated favorable performance for identifying LNM risk compared with the DH and WH approaches. In contrast, in rectal cancer, the WH approach performed better than the WH‐por approach. In the colon cohort, the LNM rate in the low‐risk subgroup was 1.7%, compared with 4.1% under the JSCCR criteria. In the rectal cohort, the corresponding rates were 8.0% and 8.6%, respectively.

**Conclusions:**

A WH‐based approach improves LNM risk stratification in T1b CRC and may help identify carefully selected patients at low risk of LNM, supporting more individualized clinical decision‐making, including careful consideration of the necessity of additional surgical resection.

Trial Registration: N/A

## Graphical abstract

This study evaluates a pathology‐based refinement of lymph node metastasis risk stratification in pT1b colorectal cancer. Compared with the conventional dominant histologybased assessment, a worst histologybased approach focused on poorly differentiated adenocarcinoma, combined with submucosal invasion depth and lymphatic invasion, more effectively identified a lower‐risk colon cancer subgroup with a reduced lymph node metastasis rate.

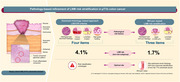

## 1 Introduction

In patients with T1 colorectal cancer (CRC), the risk of lymph node metastasis (LNM) may persist even after endoscopic resection (ER). Therefore, current clinical guidelines recommend additional surgical resection (ASR) when any histopathological risk factor (RF) for LNM is identified in the resected specimen. This approach is largely consistent across major international guidelines, including those of the National Comprehensive Cancer Network (NCCN) and the Japanese Society for Cancer of the Colon and Rectum (JSCCR). However, ≥90% of patients who undergo ASR are ultimately found to have no LNM, raising substantial concerns regarding overtreatment [1].

Recently, Zwager et al. reported that when deep submucosal invasion (DSI) is the sole RF, the observed LNM rate is as low as 2.6%. They further questioned whether this limited oncologic risk justifies routine ASR, particularly in light of surgery‐related mortality and severe complications [2]. According to the JSCCR guidelines, lesions with submucosal invasion ≥1000 µm are classified as T1b, for which ASR is principally recommended [3]. ASR is indicated when any of the following RFs are identified: poorly differentiated adenocarcinoma (por), mucinous adenocarcinoma (muc), or signet‐ring cell carcinoma (sig) as the dominant histological type (≥50% of the tumor), DSI, lymphatic invasion, venous invasion, or high‐grade tumor budding [3].

T1b CRC differs biologically from T1a CRC and more frequently harbors poorly differentiated or mucinous components [4]. Accordingly, risk assessment frameworks derived from combined T1a/T1b analyses may not fully capture pathological features specific to T1b CRC, particularly histological heterogeneity, which may influence malignant potential.

The current JSCCR guidelines rely on dominant histology (DH), defined as occupying ≥50% of the tumor. Although practical, this approach may underestimate malignant potential in T1b CRC with mixed histological features, particularly when poorly differentiated components are focal. A worst histology (WH) approach may better capture this heterogeneity and its associated malignant potential.

Therefore, we aimed to develop a T1b‐specific framework for LNM risk stratification incorporating a WH perspective in addition to conventional dominant‐type assessment. We prespecified a por‐focused worst histological model (WH‐por) using field‐based criteria within the submucosal invasive area and compared its performance with conventional JSCCR‐based stratification.

## 2 Methods

### 2.1 Study Design and Population

This retrospective, single‐center study was conducted at the Tokyo Metropolitan Cancer and Infectious Diseases Center of Komagome Hospital between April 2004 and June 2021. Only patients who had undergone surgical resection with lymph node (LN) dissection were included.

***Inclusion criteria***: (1) Pathologically confirmed T1b CRC, (2) surgical resection with LN dissection, and (3) available LN status.

***Exclusion criteria***: Hereditary CRC syndromes, synchronous advanced CRC, or prior neoadjuvant therapy.

### 2.2 Study Outcomes

The primary outcome was the LNM rate in the low‐risk subgroup. The secondary outcome was the identification of independent RFs for LNM.

### 2.3 Pathological Specimen and Standard Evaluation

#### 2.3.1 Evaluation of Primary Tumors

Specimens obtained through polypectomy, endoscopic mucosal resection, and endoscopic submucosal dissection were sliced into 2‐mm sections, whereas surgically resected specimens were sliced into approximately 5‐mm sections in accordance with routine pathological practice.

Submucosal invasion depth was measured from the muscularis mucosa, or from an estimated line when disrupted; if neither was identifiable, the distance from the mucosal surface to the deepest point of invasion was used.

Elastica van Gieson and D2‐40 immunohistochemical staining were used to evaluate venous and lymphatic invasions, respectively. Tumor budding was assessed at the invasive front and graded according to established criteria, with grade 1 considered low‐grade and grades 2–3 considered high‐grade [5].

#### 2.3.2 Evaluation of LN

All dissected LNs were bisected and examined using hematoxylin and eosin staining to assess LNM. Histological subtypes in metastatic LNs were reviewed and compared with those of the corresponding primary tumors. Archived tissue blocks were re‐sectioned when necessary.

### 2.4 Pathologist Review Process

All histological assessments of the primary tumors were performed under blinded conditions with respect to LN status and clinical outcomes. A board‐certified pathologist initially reviewed all primary tumor specimens. After a washout period of ≥1 year, the primary tumor specimens were independently re‐evaluated by a board‐certified gastrointestinal pathologist with >20 years of experience. In cases with discrepant diagnoses of the primary tumor histology (8/488, 1.6%), after a washout period of ≥6 months, the final diagnosis was made through consensus across the three independent readings to maximize reproducibility and minimize recall bias.

#### 2.4.1 Histological Type Evaluation and Definitions

Histological type was assessed using the following three approaches.

**FIGURE 1:**
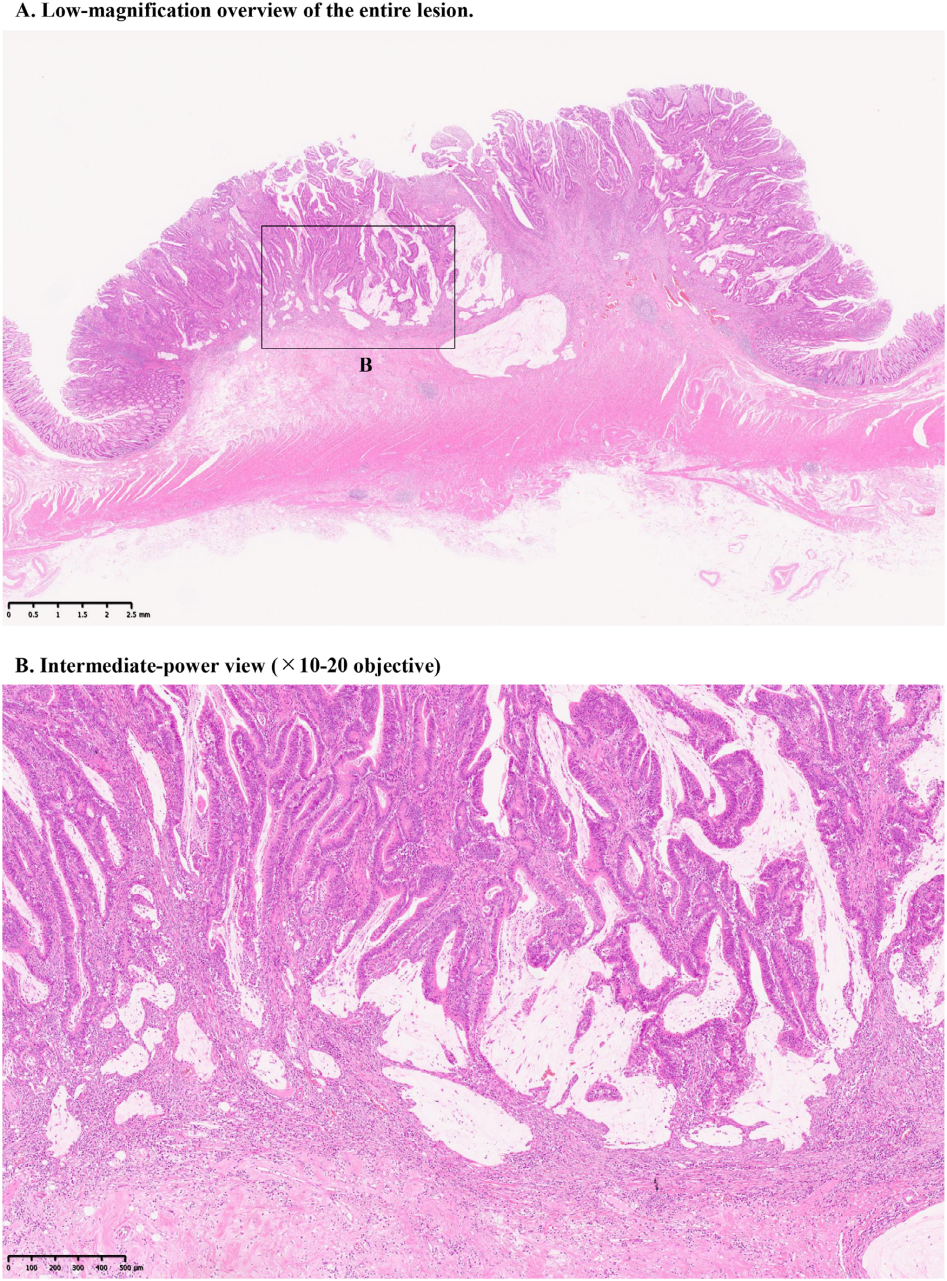
Representative histological findings illustrating the evaluation of mucinous components in the worst‐histology (WH) approach. (A) Low‐magnification overview of the entire lesion. (B) Intermediate‐power view (×10–20) of the submucosal invasive area showing extracellular mucin pools with associated tumor cells within the submucosa. In the WH evaluation, poorly differentiated adenocarcinoma (por), mucinous adenocarcinoma (muc), or signet‐ring cell carcinoma (sig) identified anywhere within the tumor was considered to represent the worst histological component. In the present figure, extracellular mucin pools with associated tumor cells identifiable within the submucosal invasive area on low‐power examination (approximately ×4) were interpreted as evidence of mucinous differentiation. The operational definition of por used in this study is illustrated in Figures 2 and 3. Signet‐ring cell components were extremely rare in this cohort (*n* = 3) and were observed only in association with poorly differentiated components; therefore, no additional operational criteria were defined for sig. Scale bars are provided in the images.

**FIGURE 2:**
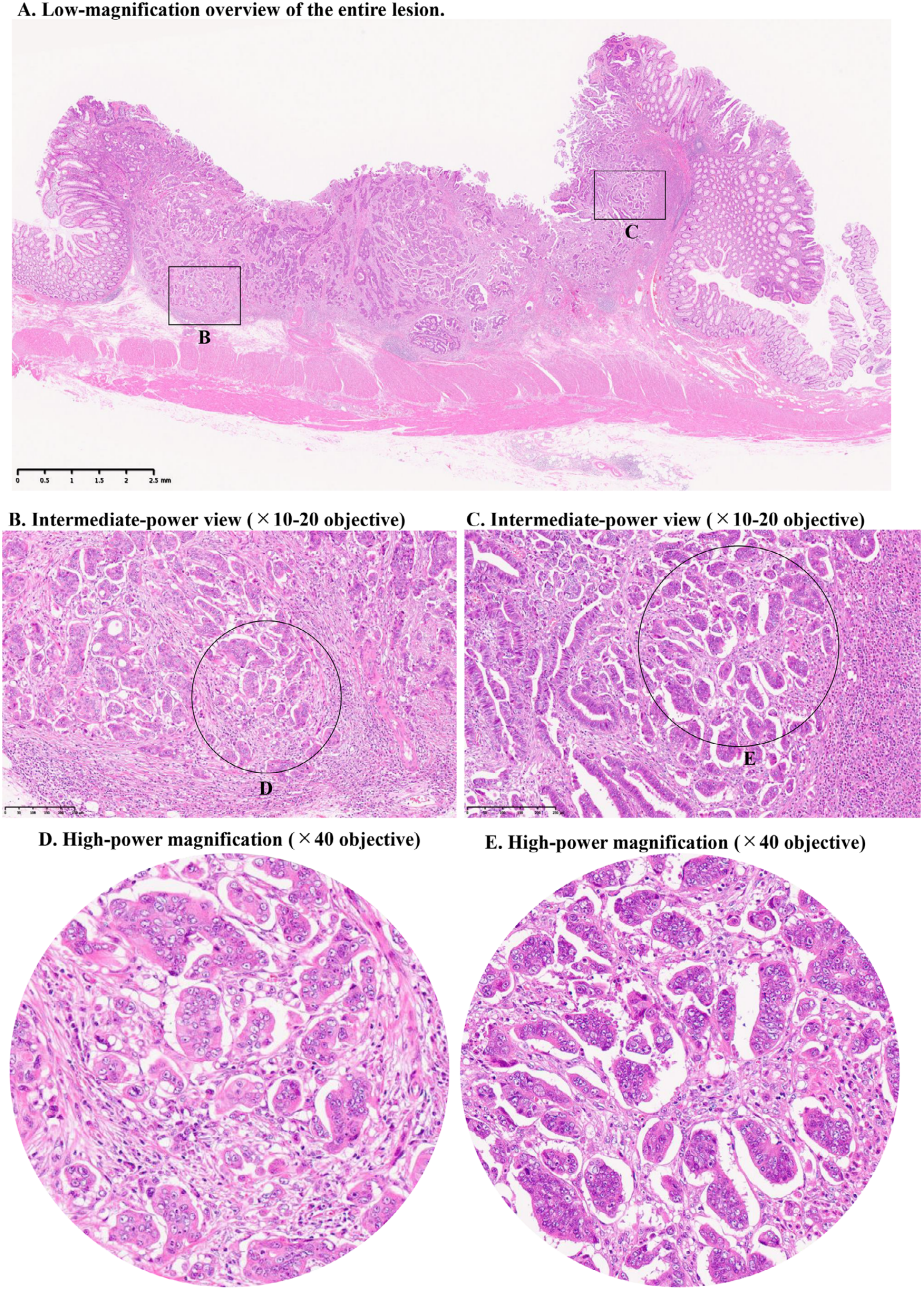
Representative histological findings illustrating a WH‐por–positive case. (A) Low‐magnification overview of the entire lesion. (B, C) Intermediate‐power views (×10–20) highlighting areas within the submucosal invasive region suspicious for poorly differentiated components. (D, E) High‐power magnification (×40). Tumor cell clusters composed of ≥5 poorly differentiated cells are observed (arrowheads), and the evaluated fields consist exclusively of poorly differentiated adenocarcinoma without gland‐forming tumor cells. These findings were reproducibly identified in ≥2 separate high‐power fields within the invasive front, fulfilling the predefined criteria for WH‐por positivity. Evaluation was performed at high‐power magnification (×40). The high‐power field corresponded to a field diameter of 0.55 mm (field number 22), with an area of approximately 0.238 mm^2^. Scale bars are provided in the images to facilitate independent interpretation.

**FIGURE 3:**
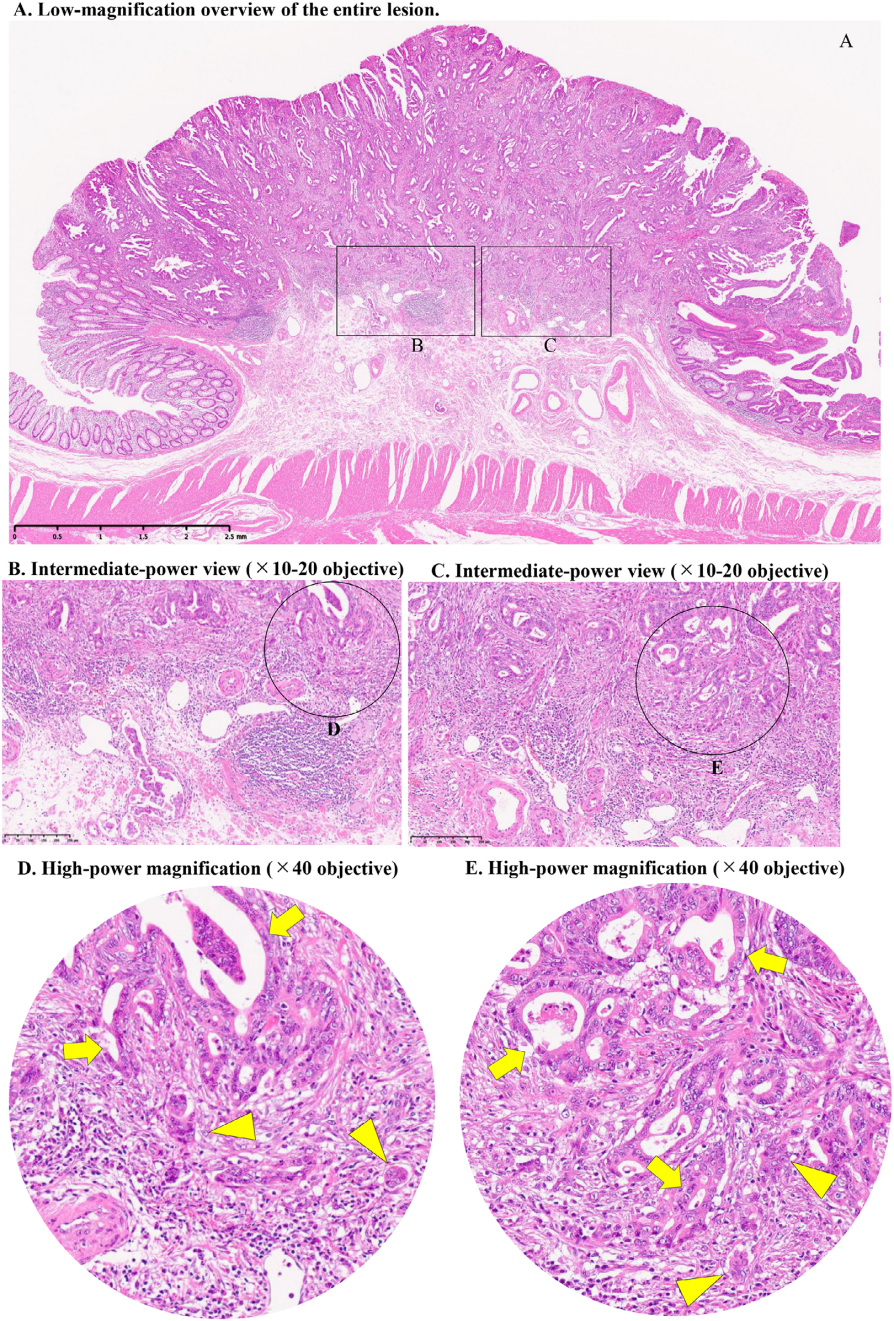
Representative histological findings illustrating a case classified as WH‐por negative despite the presence of poorly differentiated appearing tumor cells. (A) Low‐magnification overview of the entire lesion. (B, C) Intermediate‐power views (×10–20) highlighting areas within the submucosal invasive region suspicious for poorly differentiated components. (D, E) High‐power magnification (×40). Clusters of poorly differentiated‐appearing tumor cells (arrowheads) are observed; however, gland‐forming tumor cells (arrows) are also present within the same high‐power field. Because the evaluated field does not consist exclusively of poorly differentiated adenocarcinoma, the criteria for WH‐por positivity are not fulfilled. Even after evaluation of ≥2 separate high‐power fields, exclusively poorly differentiated tumor cell clusters were not reproducibly identified; therefore, this case was classified as WH‐por negative. Evaluation was performed at high‐power magnification (×40). The high‐power field corresponded to a field diameter of 0.55 mm (field number 22), with an area of approximately 0.238 mm^2^. Scale bars are provided in the images to facilitate independent interpretation.

1. DH approach: The most prevalent histological type, comprising ≥50% of the tumor, was considered dominant. Por, muc, or sig were classified as RFs when dominant. This approach is consistent with the histological evaluation recommended by the JSCCR guidelines.
2. WH approach: The histological subtype with the WH observed within the tumor was considered the worst histological component, regardless of its proportion. Por, muc, or sig were regarded as the worst histological components even when focal. Extracellular mucin pools with associated tumor cells identifiable within the submucosal invasive area on low‐power examination (×4) were interpreted as evidence of mucinous differentiation (Figure 1).
3. WH‐por approach: In the WH‐por approach, only the presence of por was considered RF. This assessment was based on the JSCCR concept of poorly differentiated components but adapted to the biological context of pT1b CRC. The entire submucosal invasive area was first screened at low magnification (×4). Areas suspicious for poorly differentiated components were then identified at intermediate magnification (×10–20) for further evaluation. Por‐positivity was then determined at high‐power magnification (×40). Por‐positivity was defined as clusters of ≥5 tumor cells lacking gland formation that were reproducibly identified in ≥2 separate high‐power fields (×40). Cellular density was not considered, but all tumor cells within the evaluated field had to consist exclusively of poorly differentiated adenocarcinoma (Figures 2 and 3).

Although extension of the WH concept to minor mucinous and signet‐ring cell components was considered, these were not included as independent variables. Signet‐ring cell carcinoma was extremely rare in this cohort (*n* = 3), and minor mucinous components were difficult to evaluate reproducibly in routine pathological practice due to variability in their extent and in the assessment of tumor differentiation within mucin pools.

### 2.5 T1b CRC Specific RFs

Univariate logistic regression analysis was performed to identify factors associated with LNM in colorectal, colon, and rectal cancers, with DH, WH, and WH‐por analyzed separately. Stepwise logistic regression based on the Akaike Information Criterion was used to identify independent RFs, with each histological model analyzed separately to avoid collinearity.

### 2.6 T1b CRC Specific LNM Risk Group Classification

Patients were stratified into low‐risk (no RFs) and high‐risk (≥1 RF) groups. Site‐specific DH‐, WH‐, and WH‐por‐based stratification models were compared with the conventional JSCCR criteria.

### 2.7 Performance Evaluation of T1b‐specific Risk Stratification Frameworks

Predictive performance was evaluated using 2×2 contingency tables, calculating sensitivity, specificity, positive predictive value, and NPV. Sensitivity and NPV were prioritized to minimize the risk of overlooking LNM.

### 2.8 Low‐risk Subgroup LNM Rate Assessment

LNM rates were calculated in low‐ and high‐risk subgroups for each framework, with low‐risk subgroup LNM rates used as the primary clinical safety indicator.

### 2.9 Clinical Acceptability Assessment of Low‐risk Subgroup LNM Rates

We prespecified three benchmarks for clinical acceptability of the low‐risk subgroup LNM rate: (1) comparison with the reported rates of severe surgical complications observed in this cohort (Clavien–Dindo grade ≥ IIIb) [6], (2) comparison with the low‐risk threshold of 2.6% established by Zwager et al., and (3) relative improvement over the conventional JSCCR**‐based** risk stratification.

### 2.10 Statistical Analysis

Categorical variables were compared using χ^2^ or Fisher's exact test, and continuous variables using Student's *t*‐test or Mann–Whitney U test, as appropriate. Cutoff values for tumor depth and size were determined using receiver operating characteristic curve analysis, selecting thresholds that ensured a sensitivity of ≥90%. All analyses were performed using EZR [7], and *p* < 0.05 was considered statistically significant.

## 3 Results

### 3.1 Patient Characteristics

**FIGURE 4:**
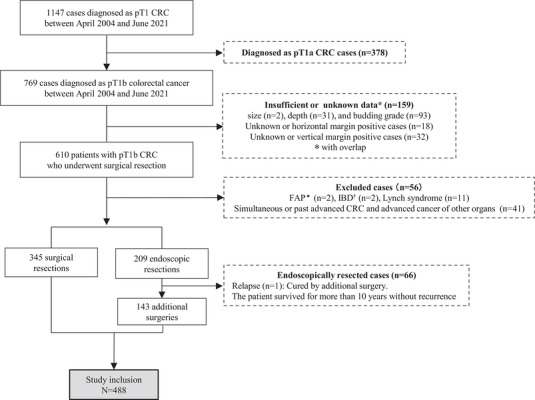
Flow diagram of patient selection for this study cohort. CRC, colorectal cancer; LNM, lymph node metastasis; FAP, familial adenomatous polyposis; IBD, inflammatory bowel disease.

A total of 488 patients with pT1b CRC (316 colon, 172 rectal) were analyzed (Figure 4). The overall LNM rate was 15.8% (77/488), with a significantly higher rate in rectal cancer than in colon cancer (22.1% vs. 12.3%, *p* = 0.006) (Table 1).

**TABLE 1:** Clinicopathological characteristics of pT1b colorectal cancer (CRC) by tumor site.

Characteristic	CRC (*n* = 488)	Colon (*n* = 316)	Rectum (*n* = 172)
**Median age** (years, range)	67 (34–90)	69 (35–90)	64 (34–90)
**Sex**			
Male	260	168	92
Female	228	148	80
**Size (mm)**			
Median (range)	18 (5–85)	18 (5–70)	20 (7–85)
**Depth (µm)**			
Median (range)	2900 (1000–16,000)	2750 (1000–16,000)	3000 (1000–10,000)
**Histological evaluation**			
**DH approach**			
Negative	483	312	171
Positive	5	4	1
**WH approach**			
Negative	417	265	152
Positive	71	51	20
**WH‐por approach**			
Negative	461	298	163
Positive	27	18	9
**Lymphatic invasion**			
Negative	340	228	112
Positive	148	88	60
**Venous invasion**			
Negative	294	199	95
Positive	194	117	77
**Tumor budding**			
Low‐grade	382	260	122
High‐grade	106	56	50
**Treatment**			
Surgical resection	345	212	133
Surgical resection after ER	143	104	39
**LNM rate (%) (n)**	15.8 (77/488)	12.3 (39/316)	22.1 (38/172)

Abbreviations: CRC, colorectal cancer; DH, dominant histology; LNM, lymph node metastasis; pT1b, pathological T1b; WH, worst histology; WH‐por, worst histology focusing on poorly differentiated adenocarcinoma.

### 3.2 Histological Evaluation of Metastatic LNs

Histological subtypes were evaluable in 74 of 77 LNM‐positive cases. Concordance between primary tumors and metastatic LNs was observed in 86.5% (64/74), whereas discordance was observed in 13.5% (10/74). In discordant cases, minor poorly differentiated components were identified in the primary tumors but did not meet predefined criteria for positivity. Detailed results are provided in Table S1 and Figure S1.

### 3.3 T1b CRC Specific RFs

#### 3.3.1 Univariate Analysis

Multiple clinicopathological factors, including histological type, lymphovascular invasion, and tumor‐related features, were significantly associated with LNM across colorectal, colon, and rectal cancers (Tables 2, 3, 4).

**TABLE 2:** Univariate analysis results of the lymph node metastasis (LNM) risk by the histological evaluation method for pT1b colorectal cancer (CRC).

Variable	LNM		Univariate analysis	
	Negative	Positive	OR (95% CI)	*p*‐Value
	(*n* = 411)	(*n* = 77)		
**Median age** (years, range)	68.0 (34–90)	64.0 (37–86)		*0.014*
**Sex**				*0.452*
Male	222	38	1.00	
Female	189	39	1.21 (0.74–1.96)	
**Location**				*0.005*
Colon	277	39	1.00	
Rectum	134	38	2.01 (1.23–3.29)	
**Type**				*0.459*
Non‐flat	216	44	1.00	
Flat	195	33	1.20 (0.74–1.97)	
**Size (mm)**				*0.041*
<13	78	7	1.00	
≥13	333	70	2.34 (1.04–5.29)	
**Invasion depth (µm)**				*0.080*
<1600	64	6	1.00	
≥1600	347	71	2.18 (0.91–5.23)	
**Histological evaluation**				
**DH approach**				*0.022*
Negative	409	74	1.00	
Positive	2	3	8.29 (1.36–50.5)	
**WH approach**				*<0.001*
Negative	366	51	1.00	
Positive	45	26	9.54 (4.23–21.50)	
**WH‐por approach**				*<0.001*
Negative	400	61	1.00	
Positive	11	16	8.04 (3.60–18.00)	
**Lymphatic invasion**				*<0.001*
Negative	310	30	1.00	
Positive	101	47	4.81(2.89–8.01)	
**Venous invasion**				*<0.001*
Negative	262	32	1.00	
Positive	149	45	2.47 (1.51–4.06)	
**Tumor budding**				*<0.001*
Low‐grade	335	47	1.00	
High‐grade	76	30	2.81 (1.67–4.74)	

Abbreviations: CI, confidence interval; CRC, colorectal cancer; DH, dominant histology; LNM, lymph node metastasis; pT1b, pathological T1b; OR, odds ratio; WH, worst histology; WH‐por, worst histology focusing on poorly differentiated adenocarcinoma.

**TABLE 3:** Univariate analysis results of the lymph node metastasis (LNM) risk by the histological evaluation method for pT1b colon cancer.

Variable	LNM		Univariate analysis	
	Negative	Positive	OR (95% CI)	*p*‐Value
	(*n* = 277)	(*n* = 39)		
**Median age** (years, range)	70.0 (35–90)	67.0 (46–86)		*0.354*
**Sex**				*0.553*
Male	149	19	1.00	
Female	128	20	1.23 (0.63–2.40)	
**Type**				*0.761*
Flat	92	12	1.00	
Non‐flat	185	27	1.12 (0.54–2.31)	
**Size (mm)**				*0.241*
<13	58	5	1.00	
≥13	219	34	1.80 (0.67–4.81)	
**Depth (µm)**				*0.018*
<2000	73	3	1.00	
≥2000	204	36	4.29 (1.28–14.40)	
**Histological evaluation**				
**DH approach**				*0.048*
Negative	275	37	1.00	
positive	2	2	7.43 (1.02–54.40)	
**WH approach**				*<0.001*
Negative	241	24	1.00	
Positive	36	15	4.18 (2.01–8.72)	
**WH‐por approach**				*<0.001*
Negative	269	29	1.00	
Positive	8	10	11.60 (4.24–31.70)	
**Lymphatic invasion**				*<0.001*
Negative	215	13	1.00	
Positive	62	26	6.94 (3.36–14.30)	
**Venous invasion**				*0.009*
Negative	182	17	1.00	
Positive	95	22	2.48 (1.26–4.89)	
**Tumor budding**				*0.002*
Low‐grade	235	25	1.00	
High‐grade	42	14	3.13 (1.51–6.51)	

Abbreviations: CI, confidence interval; DH, dominant histology; LNM, lymph node metastasis; OR, odds ratio; pT1b, pathological T1b; WH, worst histology; WH‐por, worst histology focusing on poorly differentiated adenocarcinoma.

**TABLE 4:** Univariate analysis results of the lymph node metastasis (LNM) risk by the histological evaluation method for pT1b rectal cancer.

Variable	LNM		Univariate analysis	
	Negative	Positive	OR (95% CI)	*p*‐Value
	(*n* = 134)	(*n* = 38)		
**Median age** (years, range)	65.0 (34–90)	60.0 (37–78)		*0.048*
**Sex**				*0.625*
Male	73	19	1.00	
Female	61	19	1.20 (0.58–2.46)	
**Type**				*0.010*
Non‐flat	103	21	1.00	
Flat	31	17	2.69 (1.26–5.72)	
**Size (mm)**				*0.470*
<16	36	8	1.00	
≥16	98	30	1.38 (0.58–3.28)	
**Depth (µm)**				*0.560*
<1500	15	3	1.00	
≥1500	119	35	1.47 (0.40–5.37)	
**Histological evaluation**				
**DH approach**				*0.986*
Negative	134	37	1.00	
Positive	0	1	7,670,000 (0–Inf)	
**WH approach**				*<0.001*
Negative	125	27	1.00	
Positive	9	11	5.66 (2.14–15.00)	
**WH‐por approach**				*0.004*
Negative	131	32	1.00	
Positive	3	6	8.19 (1.94–34.50)	
**Lymphatic invasion**				*0.004*
Negative	95	17	1.00	
Positive	39	21	3.01 (1.44–6.31)	
**Venous invasion**				*0.029*
Negative	80	15	1.00	
Positive	54	23	2.27 (1.09–4.74)	
**Tumor budding**				*0.048*
Low‐grade	100	22	1.00	
High‐grade	34	16	2.14 (1.01–4.54)	

Abbreviations: LNM, lymph node metastasis; pT1b, pathological T1b; OR, odds ratio; CI, confidence interval; DH, dominant histology; WH, worst histology; WH‐por, worst histology focusing on poorly differentiated adenocarcinoma.

#### 3.3.2 Multivariate Analysis

Histological type remained an independent predictor of LNM in multiple models. Lymphatic invasion was consistently identified as an independent RF, while additional factors varied by tumor location (Table 5).

**TABLE 5:** Multivariate analysis of lymph node metastasis (LNM) risk factors in pT1b colorectal cancer (CRC) by each histological evaluation stratified by tumor site.

	DH approach		WH approach		WH‐por approach	
Variable	OR (95%CI)	*p*‐value	OR (95%CI)	*p*‐value	OR (95%CI)	*p*‐value
**A) CRC**						
Rectum	1.85 (1.09–3.13)	*0.022*	2.04 (1.19–3.51)	*0.010*	1.94 (1.13–3.32)	*0.017*
Size ≥13mm	2.13 (0.90–5.01)	*0.084*	1.99 (0.84–4.72)	*0.117*	2.36 (0.98–5.69)	*0.056*
**Histological type**	**11.70 (1.66–82.30)**	***0.014***	**3.87 (2.08–7.20)**	***<0.001***	**7.10 (2.87–17.60)**	***<0.001***
Lymphatic invasion‐positive	4.11 (2.41–6.99)	*<0.001*	3.60 (2.09–6.18)	*<0.001*	3.41 (1.98–5.89)	*<0.001*
Venous invasion‐positive	1.89 (1.11–3.21)	*0.020*	1.93 (2.08–7.20)	*<0.001*	1.71 (0.99–2.95)	*0.053*
High‐grade tumor budding	—	—	—	—	—	—
**B) Colon cancer**						
Depth ≥2000µm	3.70 (1.07–12.80)	*0.039*	3.83 (1.07–13.70)	*0.038*	5.30 (1.36–20.60)	*0.016*
**Histological type**	5.34 (0.58–49.00)	*0.138*	**3.13 (1.40 –6.99)**	***0.005***	**8.15 (2.66–25.00)**	*<* ***0.001***
Lymphatic invasion‐positive	6.59 (3.16–13.70)	*<0.001*	5.21 (2.44–11.20)	*<0.001*	5.40 (2.52–11.60)	*<0.001*
Venous invasion‐positive	—	—	1.84 (0.87–3.90)	*0.112*	—	—
High‐grade tumor budding	—	—	—	—	—	—
**C) Rectal cancer**						
Flat type			2.92 (1.28–6.65)	*0.011*	2.45 (1.10–5.45)	*0.028*
**Histological type**			**6.03 (2.10–17.30)**	***<0.001***	**6.30 (1.35–29.5)**	***0.019***
Lymphatic invasion‐positive			2.62 (1.19–5.76)	*0.017*	2.56 (1.18–5.56)	*0.018*
Venous invasion‐positive			—	—	—	—
High‐grade tumor budding	—	—	—	—	—	—

*Note*: Bold text denotes histological subtypes identified as significant risk factors; gray shading indicates risk factors that were significant when those subtypes were applied.

Abbreviations: CI, confidence interval; CRC, colorectal cancer; DH, dominant histological differentiation; LNM, lymph node metastasis; OR, odds ratio; pT1b, pathological T1b; WH, worst histological differentiation; WH‐por, worst histology focusing on poorly differentiated adenocarcinoma.

#### 3.3.3 Predictive Performance Analysis and Low‐risk Subgroup LNM Rate Assessment

Histology‐based risk stratification models demonstrated higher sensitivity than the JSCCR criteria in colorectal and colon cancer analyses, whereas this advantage was not observed in rectal cancer.

**FIGURE 5:**
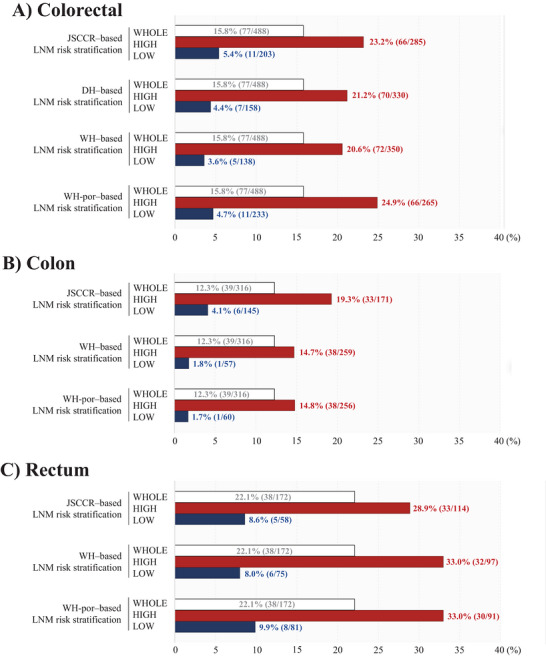
Comparison of LNM rates in the low‐risk LNM subgroups identified by different risk stratification frameworks for colorectal cancer (A), colon cancer (B), and rectal cancer (C). Numbers in parentheses indicate the number of patients with LNM and the total number of patients in each group. LNM, lymph node metastasis.

In CRC overall, the WH‐por‐based model achieved the best performance (sensitivity 93.5%, NPV 96.4%) (Table 6A). The low‐risk subgroup LNM rate decreased from 5.4% (JSCCR) to 3.6% (WH‐based model) (Figure 5A).

**TABLE 6:** Comparison of the predictive performance between the Japanese Society for Cancer of the Colon and Rectum (JSCCR) and novel frameworks.

Stratification	RFs	Sensitivity	Specificity	PPV	NPV
**A) Colorectal cancer**					
JSCCR‐based	Histological type, ly, v, Budding	0.857	0.467	0.232	0.946
DH‐based	Rectum, Histological type, ly, v	0.909	0.367	0.212	0.956
WH‐based	Rectum, Histological type, ly, v	0.935	0.324	0.206	0.964
WH‐por‐based	Rectum, Histological type, ly	0.857	0.516	0.249	0.951
**B) Colon cancer**					
JSCCR‐based	Histological type, ly, v, Budding	0.846	0.502	0.193	0.959
WH‐based	Depth (≥2000µm), Histological type, ly	0.974	0.202	0.147	0.982
WH‐por‐based	Depth (≥2000µm), Histological type, ly	0.974	0.213	0.148	0.983
**C) Rectal cancer**					
JSCCR‐based	Histological type, ly, v, Budding	0.868	0.396	0.289	0.914
WH‐based	Flat type, Histological type, ly	0.842	0.515	0.330	0.920
WH‐por‐based	Flat type, Histological type, ly	0.789	0.545	0.330	0.901

Abbreviations: DH, dominant histology; JSCCR, Japanese Society for Cancer of the Colon and Rectum; ly, lymphatic invasion; NPV, negative predictive value; PPV, positive predictive value; RF, risk factor; v, venous invasion; WH, worst histology; WH‐por, worst histology focusing on poorly differentiated adenocarcinoma.

In colon cancer, the WH‐por‐based model showed the best performance (sensitivity 97.4%, NPV 98.3%) (Table 6B), with a low‐risk subgroup LNM rate of 1.7% compared with 4.1% using JSCCR criteria (Figure 5B).

In rectal cancer, the WH‐based model showed the most balanced performance (NPV 92.0%) (Table 6C), with a low‐risk subgroup LNM rate of 8.0% compared with 8.6% using JSCCR criteria (Figure 5C).

#### 3.3.4 Clinical Acceptability Assessment of Low‐risk Subgroup LNM Rates

The observed LNM rates in the colon low‐risk subgroup (1.7–1.8%) were lower than those with conventional JSCCR criteria, below the 2.6% threshold proposed by Zwager et al., and comparable to the rate of severe surgical complications in our cohort (Clavien–Dindo grade ≥ IIIb, 1.3%).

#### 3.3.5 Proposed Pathology‐based Framework for Identifying a Low‐risk Subgroup of pT1b Colon Cancer After Endoscopic Resection

**FIGURE 6:**
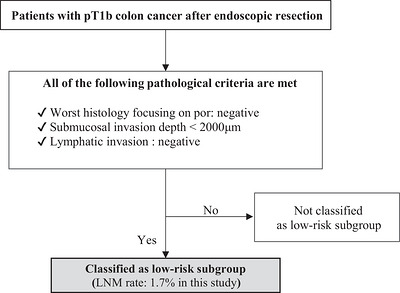
Proposed pathology‐based framework for identifying a low‐risk subgroup of pT1b colon cancer after endoscopic resection. This flowchart illustrates a criterion‐based approach using routinely assessable pathological factors to identify a subgroup of patients with pT1b colon cancer who exhibited a very low observed risk of lymph node metastasis (LNM) in the present study. Patients who met all of the following criteria were classified as the low‐risk subgroup: WH‐por negativity, submucosal invasion depth <2000 µm, and absence of lymphatic invasion. The observed LNM rate in this subgroup was 1.7% (1/60). This framework is intended to support risk stratification and individualized clinical decision‐making and should not be interpreted as a recommendation to omit additional surgical resection.

In pT1b colon cancer, patients with WH‐por negativity, submucosal invasion depth <2000 µm, and absence of lymphatic invasion were classified as low risk (Figure 6).

## 4 Discussion

In this study, we developed a T1b‐specific histological risk stratification framework incorporating a WH approach. Compared with conventional DH‐based assessment, this framework enabled more refined risk stratification, particularly in colon cancer. In the colon cohort, the WH‐por‐based model identified a low‐risk subgroup with a very low LNM rate of 1.7%, lower than that observed using the conventional JSCCR criteria (4.1%). Importantly, this framework achieved high sensitivity for LNM detection using fewer routinely assessed pathological factors, supporting its potential clinical applicability in routine pathological practice.

A key biological premise underlying this study is the marked histological heterogeneity observed in pT1b CRC. Early invasive tumors may harbor minor poorly differentiated components even when the predominant histology appears well or moderately differentiated [4]. Because conventional DH‐based evaluation requires a histological subtype to occupy ≥50% of the tumor, it may underestimate malignant potential when such components are present only focally. By prioritizing the most aggressive component regardless of its proportion, the WH‐based approach may better capture this heterogeneity. Although prior studies have suggested the prognostic relevance of minor poorly differentiated components in early CRC [8], a reproducible framework for their evaluation in routine practice has not been established. In this study, we operationalized this concept by focusing on reproducible identification of poorly differentiated components within the submucosal invasive area.

To ensure reproducibility, we developed an operational definition for poorly differentiated components adapted from, but distinct from, the JSCCR criteria. While JSCCR defines poorly differentiated adenocarcinoma based on the largest area occupying a high‐power field (×40), this approach may lead to overinterpretation in pT1b CRC, where such components are often sparse and scattered within abundant submucosal stroma. In pT1b CRC, sectioning artifacts may occasionally produce structures that mimic poorly differentiated morphology, such as tangentially sectioned or truncated glands appearing as non‐gland‐forming clusters. Therefore, rather than relying on a single high‐power field, we first screened the entire submucosal invasive area and restricted high‐power evaluation to candidate regions. To minimize incidental findings and ensure reproducibility, positivity required consistent identification across multiple fields. This approach also differs fundamentally from the poorly differentiated cluster (PDC) grading system [9]. While both approaches consider clusters of ≥5 tumor cells with poor gland formation, PDC represents a grading concept based on tumor architecture rather than a histological subtype. In addition, PDC is defined by clusters with clearly defined borders, regardless of their location inside or outside lymphovascular structures. In contrast, the WH‐por approach evaluates poorly differentiated adenocarcinoma as a histological component within the submucosal invasive area using a field‐based assessment. These differences reflect distinct pathological concepts and evaluation frameworks.

Comparison of histological subtypes between primary tumors and metastatic LNs demonstrated that the worst histological components were reflected in metastatic lesions in most cases, although discordant patterns were also observed. In several discordant cases, very limited poorly differentiated components were identified in the primary tumor, but did not meet predefined criteria for positivity. These findings, based on direct comparison between primary tumors and corresponding metastatic lesions, suggest that even minimal poorly differentiated components may be associated with metastatic potential, supporting the biological relevance of WH‐based assessment, even when such components fall below conventional diagnostic thresholds.

When stratified by tumor location, clear differences were observed between colon and rectal cancer. In colon cancer, WH‐por negativity combined with shallow submucosal invasion and absence of lymphatic invasion identified a subgroup with a very low risk of LNM. In contrast, the performance of histology‐based stratification was less favorable in rectal cancer, where low‐risk subgroup LNM rates remained relatively high. These differences may reflect anatomical and biological distinctions, including variations in lymphatic drainage patterns and tumor microenvironment, as well as potential differences in clinical and pathological practices [10, 11, 12]. These findings suggest that the applicability of histology‐based risk stratification may be site‐dependent and should be interpreted cautiously in rectal cancer.

From a clinical perspective, the motivation for refining risk stratification in pT1b CRC lies in balancing oncologic safety against overtreatment, a dilemma increasingly emphasized in recent literature [13]. Although ASR is recommended when any histopathological RF is present, most patients ultimately have no LNM. Moreover, accumulating evidence highlights the non‐negligible morbidity and mortality associated with surgery [2, 14]. In this study, the LNM rate in the colon low‐risk subgroup identified by the WH‐por‐based framework (1.7%) was close to the rate of severe postoperative complications (Clavien–Dindo grade ≥IIIb, 1.3%) observed in this cohort. These findings support the potential role of refined pathological assessment in enabling more individualized clinical decision‐making, including careful consideration of the necessity of ASR, particularly in carefully selected patients.

This study has several limitations. First, this was a retrospective single‐center study, which may limit generalizability, although the standardized re‐review process may have contributed to consistency in histological assessment. Second, long‐term oncological outcomes were not evaluated. Third, pathological assessment may not detect all microscopic disease, and therefore, the absence of LNM on histology does not exclude minimal residual disease. Accordingly, this framework should be used to identify a subgroup with a very low observed risk rather than to exclude nodal metastasis.

Despite these limitations, our findings highlight the importance of reproducible evaluation of the worst histological components in pT1b CRC. This framework provides a practical approach to improve risk stratification beyond predominant histology and support individualized clinical decision‐making, particularly in colon cancer. Further external validation is warranted before clinical implementation.

## 5 Conclusion

A WH‐based approach may improve LNM risk stratification in T1b CRC. In colon cancer, this framework identified a subgroup with a very low observed risk of LNM (1.7%), supporting more individualized post‐endoscopic management, including careful consideration of the necessity of ASR. Further validation in independent cohorts is warranted.

## Author Contributions

**Ryoko Shimizuguchi**: investigation and validation. **Koichi Koizumi**: conceptualization, methodology, investigation, validation, and supervision. **Satomi Shibata**: conceptualization, methodology, writing – original draft, writing – review and editing, project administration, investigation, data curation, and validation. **Shin‐ichiro Horiguchi**: visualization, validation, methodology, and supervision. **Daichi Sadato**: software, data curation, formal analysis, and visualization. **Misato Takao**: investigation, validation, and resources. **Jun Nakahodo**: investigation, validation, and writing – review and editing. **Hiroki Kato**: investigation, validation, and resources. **Kazushige Kawai**: investigation, validation, resources, and writing – review and editing. **Akinari Takao**: investigation and validation. **Itaru Kubota**: investigation, validation, and resources. **Tsunekazu Hishima**: investigation, validation, and resources. **Tatsuro Yamaguchi**: investigation, validation, and writing – review and editing. **Toshiro Iizuka**: supervision, writing – review and editing, and project administration. **Daisuke Nakano**: investigation, validation, and resources. **Yawara Kubota**: investigation, validation, and resources.

## Funding

The authors have nothing to report.

## Ethics Statement

This retrospective study was conducted in accordance with the ethical principles outlined in the Declaration of Helsinki and was approved by the Institutional Review Board of the Tokyo Metropolitan Cancer and Infectious Diseases Center at Komagome Hospital (approval number: 2918).

## Consent

Individual patient consent was waived owing to the retrospective nature of the study and the use of anonymized data. An opt‐out clause was included to ensure patient privacy and confidentiality.

## Conflicts of Interest

The authors declare no conflicts of interest.

## Artificial Intelligence

Not applicable.

## Supporting information




**Figure S1**: Concordance of worst histology between primary tumors and metastatic lymph nodes in patients with LNM. Among 77 cases with LNM, histological comparison was possible in 74 cases. Three cases were unevaluable because of tissue exhaustion (*n* = 2) or the presence of only a single indeterminate tumor cell cluster (*n* = 1). Overall, concordance between primary tumors and metastatic LNs was observed in 86.5% of evaluable cases, whereas discordance was observed in 13.5%. The table summarizes the relationship between the worst histological components in the primary tumors and those identified in metastatic LNs. Tub/pap indicates differentiated adenocarcinoma (tubular or papillary type). LN, lymph node; LNM, lymph node metastasis.

**Table S1** Case‐by‐case comparison of worst histology between primary tumors and metastatic LNs in 77 patients with LNM.
This table provides a detailed case‐by‐case comparison of the worst histological components between primary tumors and corresponding metastatic LNs in 77 patients with LNM. Concordance was defined as agreement in the worst histology between the primary tumor and metastatic LN. Histological comparison was not feasible in unevaluable cases due to tissue exhaustion or the presence of only a single indeterminate tumor cell cluster. NA indicates not available. Gray shading highlights discordant cases.

## Data Availability

Deidentified clinical data underlying this article are not publicly available due to institutional and ethical restrictions. However, the datasets may be shared by the corresponding author upon reasonable request and with approval from the institutional review board.
