# Supplementary material for: Worst Histology‐Based Risk Stratification for Lymph Node Metastasis in Patients With T1b Colorectal Cancer: A Retrospective Pathology‐based Study
Source: DEN Open. 2026 Jun 4;7(1):e70355. doi: 10.1002/deo2.70355 (PMC13238743; doi:10.1002/deo2.70355)
Supplement: Supplementary file 1 — Figure S1: Concordance of worst histology between primary tumors and metastatic lymph nodes in patients with LNM. Among 77 cases with LNM, histological comparison was possible in 74 cases. Three cases were unevaluable because of tissue exhaustion (n = 2) or the presence of only a single indeterminate tumor cell cluster (n = 1). Overall, concordance between primary tumors and metastatic LNs was observed in 86.5% of evaluable cases, whereas discordance was observed in 13.5%. The table summarizes the relationship between the worst histological components in the primary tumors and those identified in metastatic LNs. Tub/pap indicates differentiated adenocarcinoma (tubular or papillary type). LN, lymph node; LNM, lymph node metastasis. [file DEO2-7-e70355-s002.pptx]

## Slide 1
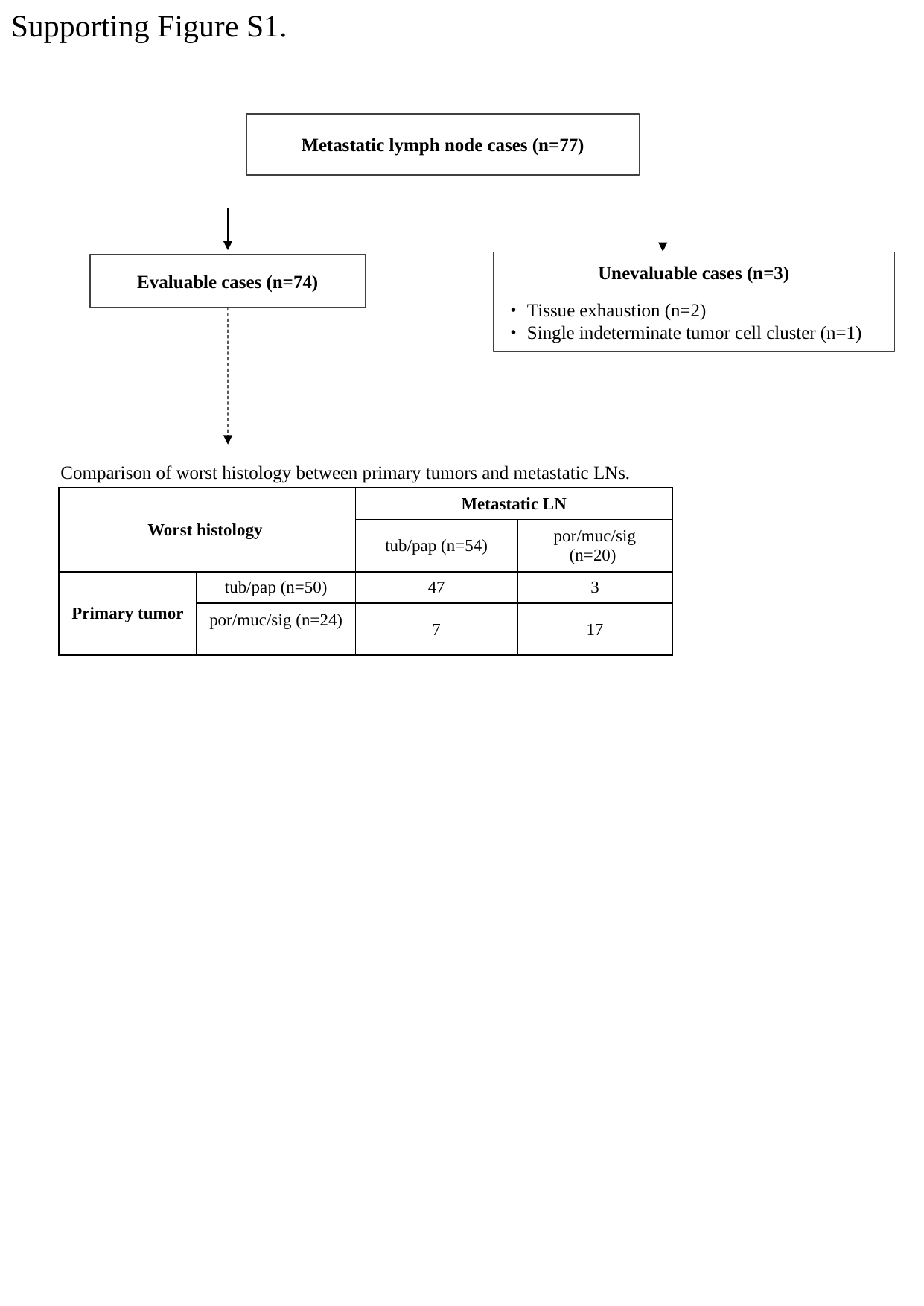

Supporting Figure S1.
Metastatic lymph node cases (n=77)
Unevaluable cases (n=3)
・Tissue exhaustion (n=2)
・Single indeterminate tumor cell cluster (n=1)
Evaluable cases (n=74)
Comparison of worst histology between primary tumors and metastatic LNs.
| Worst histology | | Metastatic LN | |
| --- | --- | --- | --- |
| | | tub/pap (n=54) | por/muc/sig (n=20) |
| Primary tumor | tub/pap (n=50) | 47 | 3 |
| | por/muc/sig (n=24) | 7 | 17 |
