# Supplementary material for: Worst Histology‐Based Risk Stratification for Lymph Node Metastasis in Patients With T1b Colorectal Cancer: A Retrospective Pathology‐based Study
Source: DEN Open. 2026 Jun 4;7(1):e70355. doi: 10.1002/deo2.70355 (PMC13238743; doi:10.1002/deo2.70355)
Supplement: Supplementary file 2 — Table S1 Case‐by‐case comparison of worst histology between primary tumors and metastatic LNs in 77 patients with LNM. This table provides a detailed case‐by‐case comparison of the worst histological components between primary tumors and corresponding metastatic LNs in 77 patients with LNM. Concordance was defined as agreement in the worst histology between the primary tumor and metastatic LN. Histological comparison was not feasible in unevaluable cases due to tissue exhaustion or the presence of only a single indeterminate tumor cell cluster. NA indicates not available. Gray shading highlights discordant cases. [file DEO2-7-e70355-s001.docx]

**Supporting Table S1. Case-by-case comparison of worst histology between primary tumors and metastatic LNs in77 patients with LNM.**

| Case No. | Location | Worst histology in primary tumor | Worst histology in metastatic LN | Concordance  (Y/N) |
| --- | --- | --- | --- | --- |
| 1 | colon | tub | tub | Y |
| 2 | colon | tub | tub | Y |
| 3 | colon | tub | tub | Y |
| 4 | colon | tub | tub | Y |
| 5 | colon | muc | muc | Y |
| 6 | colon | muc | muc | Y |
| 7 | colon | tub | tub | Y |
| 8 | colon | tub | tub | Y |
| 9 | colon | tub | tub | Y |
| 10 | colon | tub | tub | Y |
| 11 | colon | tub | tub | Y |
| 12 | colon | tub/pap | tub | Y |
| 13 | colon | muc | muc | Y |
| 14 | colon | tub | tub | Y |
| 15 | colon | tub | tub | Y |
| 16 | colon | tub | tub | Y |
| 17 | colon | tub | tub | Y |
| 18 | colon | por | por | Y |
| 19 | colon | tub | tub | Y |
| 20 | colon | por | por | Y |
| 21 | colon | tub | tub | Y |
| 22 | colon | tub | tub | Y |
| 23 | colon | por/muc | muc | Y |
| 24 | colon | tub | tub | Y |
| 25 | colon | por | por | Y |
| 26 | colon | tub | tub | Y |
| 27 | colon | muc | muc | Y |
| 28 | colon | tub | tub | Y |
| 29 | colon | tub | tub | Y |
| 30 | colon | por | por | Y |
| 31 | colon | muc | muc/por | Y |
| 32 | colon | tub | Unevaluable* | NA |
| 33 | colon | por | Unevaluable* | NA |
| 34 | colon | tub | por | N |
| 35 | colon | por | tub | N |
| 36 | colon | por/muc | tub | N |
| 37 | colon | tub | muc | N |
| 38 | colon | por | tub | N |
| 39 | colon | por | tub | N |
| 40 | rectum | tub | tub | Y |
| 41 | rectum | tub | tub | Y |
| 42 | rectum | tub | tub | Y |
| 43 | rectum | tub | tub | Y |
| 44 | rectum | tub | tub | Y |
| 45 | rectum | tub | tub | Y |
| 46 | rectum | tub | tub | Y |
| 47 | rectum | muc | muc | Y |
| 48 | rectum | tub | tub | Y |
| 49 | rectum | tub | tub | Y |
| 50 | rectum | por | por | Y |
| 51 | rectum | por | por | Y |
| 52 | rectum | muc | muc | Y |
| 53 | rectum | tub | tub | Y |
| 54 | rectum | tub | tub | Y |
| 55 | rectum | tub | tub | Y |
| 56 | rectum | por/muc | por | Y |
| 57 | rectum | tub | tub | Y |
| 58 | rectum | tub/pap | tub | Y |
| 59 | rectum | tub | tub | Y |
| 60 | rectum | tub | tub | Y |
| 61 | rectum | tub | tub | Y |
| 62 | rectum | tub | tub | Y |
| 63 | rectum | tub | tub | Y |
| 64 | rectum | tub | tub | Y |
| 65 | rectum | tub | tub | Y |
| 66 | rectum | tub | tub | Y |
| 67 | rectum | tub | tub | Y |
| 68 | rectum | tub | tub | Y |
| 69 | rectum | tub | tub | Y |
| 70 | rectum | tub | tub | Y |
| 71 | rectum | muc | muc | Y |
| 72 | rectum | muc | muc | Y |
| 73 | rectum | por/muc/sig | Unevaluable† | NA |
| 74 | rectum | tub | por | N |
| 75 | rectum | por | tub | N |
| 76 | rectum | muc | tub | N |
| 77 | rectum | por | tub | N |

Y, yes; N, no; NA, not available; LN, lymph node; LNM, lymph node metastasis

＊tissue exhaustion; † single indeterminate tumor cell cluster

Gray shading indicates discordant cases.
